# Variations in Ecological Locations Induce Soybean Seed Wrinkles by Disrupting Source–Sink Relationship and Energy Metabolism at the Grain-Filling Stage

**DOI:** 10.3390/plants15121924

**Published:** 2026-06-22

**Authors:** Junxia Huang, Wei Zheng, Demin Rao, Xingdong Yao, Futi Xie, Huijun Zhang, Xue Ao, Haiying Wang, Yongqiang Cao

**Affiliations:** 1 Soybean Research Institute, Shenyang Agricultural University, Shenyang 110866, China; hjx11150906@163.com (J.H.); xingdongyao@syau.edu.cn (X.Y.); xft299@syau.edu.cn (F.X.); 1991500012@syau.edu.cn (H.Z.); a2009syau@syau.edu.cn (X.A.); 2 Jiamusi Branch of Heilongjiang Academy of Agricultural Sciences, Jiamusi 154007, China; zhw105122@163.com; 3 Institute of Soybean Research, Jilin Academy of Agricultural Sciences (Northeast Agricultural Research Center of China), Changchun 130033, China; rdm1397155464@163.com; 4 Crop Research Institute, Liaoning Academy of Agricultural Sciences, Shenyang 110866, China

**Keywords:** soybean, different ecological locations, source–sink relationship, energy metabolism, dry matter translocation, seed wrinkling, short-term high temperature

## Abstract

Defective seed filling, which manifests as seed wrinkling, severely impairs the yield and commercial quality of soybean crops. Soybean varieties independently developed in Heilongjiang Province exhibit distinct phenotypic variations in seed wrinkling across diverse ecological planting regions, whereas the molecular and physiological mechanisms driving such differences remain largely uncharacterized. In this study, two soybean genotypes with divergent heat resistance, namely, the heat-sensitive cultivar HH43 and the heat-tolerant cultivar HN76, were planted in three distinct ecological sites for comparative analysis. Statistical results indicated that ecological conditions serve as the predominant factor regulating seed-wrinkling variation, with high temperatures occurring during the seed-filling stage identified as the key abiotic stress trigger. Excessively high ambient temperatures triggered abnormal sucrose accumulation in the pod husks of heat-vulnerable HH43, disrupting the coupling relationship between sucrose metabolism and energy supply and thereby restricting starch biosynthesis in developing seeds. Transcriptome profiling combined with weighted gene co-expression network analysis (WGCNA) further demonstrated that heat stress significantly suppressed the expression of energy transport-related genes and induced the dysregulated expression of starch synthesis-associated genes in susceptible soybean plants, and these transcriptional alterations were further verified via qRT-PCR assays. Collectively, short-term extreme high temperatures interrupt the carbon transport and allocation process from pod husks to seeds in heat-sensitive soybean cultivars. By contrast, heat-tolerant genotypes can sustain a stable physiological metabolism and molecular regulatory networks to effectively cope with high-temperature stress during the seed-filling period.

## 1. Introduction

Soybean (*Glycine max*) acts as a significant grain and oil crop with significant economic worth [1]. As China’s economy advances rapidly and residents’ quality of life improves gradually in recent years, the market demand for soybeans has continued on an uptrend. Furthermore, soybean serves a fundamental role in safeguarding China’s edible oil supply security [2]. Seed wrinkling, alternatively termed “filling obstruction,” is defined as the failure of seeds to achieve regular plumpness during maturation, leading to rough seed coats and irregular morphologies. This phenotypic flaw is widespread in crop breeding (e.g., soybean, rice, wheat and other crops) [3,4,5,6,7,8,9,10]. Soybean undergoes multiple growth and development stages during its life cycle. Among these stages, the seed-filling stage is a critical period that directly affects crop yield and quality [11,12], and it is also the sole key stage for seed-filling and development. Sufficient seed filling is a prerequisite for plump and round seeds [13]. During the seed-filling stage, the vegetative organs of soybean tend to cease, while the assimilate transport system within the plant remains highly active. A large quantity of photosynthates and mineral elements stored in vegetative organs in the early stage are translocated to pods and seeds [14,15,16]. This stage contributes the most significant increase in dry matter throughout the plant’s life cycle, and the smoothness of its progression directly determines the final plumpness of seeds [17]. In recent years, short-term extreme high temperatures have occurred frequently in major soybean-producing regions against the backdrop of global warming; given that the seed-filling stage is extremely sensitive to temperature stress, high temperatures during this period have become the primary climatic factor inducing seed wrinkling [18,19].

The quality of seed development depends on the coordinated operation of the ‘source-sink-flow’ system and the steady-state support of energy metabolism during the grain-filling stage: photosynthates synthesized in source organs (leaves) are transported to sink organs (grains) via the ‘flow’ (vascular system and other transport tissues including pod husks), while ATP generated by energy metabolism provides the driving force for substance translocation and seed filling. Ultimately, the plump development of seeds is achieved through the synthesis of starch, protein, and other substances in seeds [20,21,22]. Previous studies have demonstrated that high temperatures disrupt the source–sink balance during the grain-filling stage, reduce the translocation efficiency of assimilates, and perturb energy metabolism, ultimately leading to insufficient seed filling [23,24,25,26]. However, there are significant genotypic differences in the responses of different soybean cultivars to high-temperature stress. Under the same environmental stress, some cultivars exhibit strong tolerance, whereas sensitive cultivars show severe stress responses [27,28,29]. The internal mechanism underlying the differences in seed development induced by this ‘cultivar × environment’ interaction remains unclear. In particular, research on the coupling regulation between the transport function of “flow” organs (pod husks) and energy metabolism is still scarce.

China has a vast span of soybean-planting ecological locations. Due to latitudinal differences, there are significant differences in the temperature rhythm and extreme high temperature occurrence characteristics during the grain-filling stage between the Sanjiang Plain in Northeast China (e.g., Jiamusi) and the Liaohe Plain (e.g., Shenyang), which provides suitable research conditions for studying the interaction effect between cultivars and the environment [30,31]. Preliminary investigations have revealed that two soybean cultivars, HH43 and HN76, both exhibited plump seeds when grown in Jiamusi. By contrast, when cultivated in Shenyang, HH43 showed severe seed wrinkles, while HN76 maintained normal seed development. This phenomenon suggests that there may be specific environmental stress during the grain-filling stage in the Shenyang ecological location and that there are genotypic differences between the two cultivars in their responses to this stress. Preliminary investigations have revealed that the occurrence of soybean grain-filling disorders in the Sanjiang Plain (e.g., Jiamusi) and Liaohe Plain (e.g., Shenyang) of Northeast China is closely associated with the temperature differences during the grain-filling stage between the two locations and that such temperature variations constitute the core causal factor for the disorders [29]. However, the underlying mechanisms governing the occurrence of grain-filling disorders in these two regions remain unclear. Therefore, this study aimed to elucidate the formation mechanism of soybean seed wrinkles under the cultivar × environment interaction, with a specific focus on the source–sink–flow relationship and energy metabolism processes.

In this study, HH43 and HN76 were used as experimental materials. Leveraging the natural environmental differences between two major ecological locations (i.e., Jiamusi and Shenyang), multivariate analysis of variance (ANOVA) was conducted to clarify the ‘cultivar × environment’ interaction effect and key influencing factors. The functional differences of the source–sink–flow system were analyzed through the determination of physiological indicators, and whole-genome transcriptome sequencing was utilized to identify differentially expressed genes (DEGs) in pod husks and seeds. Additionally, weighted gene co-expression network analysis (WGCNA) was implemented to screen for core regulatory modules and hub genes, followed by qRT-PCR validation of the selected genes. This study’s objective is to systematically elucidate the formation mechanism of soybean seed wrinkles under the ‘cultivar × environment’ interaction, thereby providing a theoretical groundwork for the breeding of high-temperature-resistant and wrinkle-resistant soybean cultivars as well as the implementation of ecological adaptive cultivation.

## 2. Results

### 2.1. Phenotypic Variation in Soybean Seed Shriveling and Its Key Environmental Drivers

**Figure 1:**
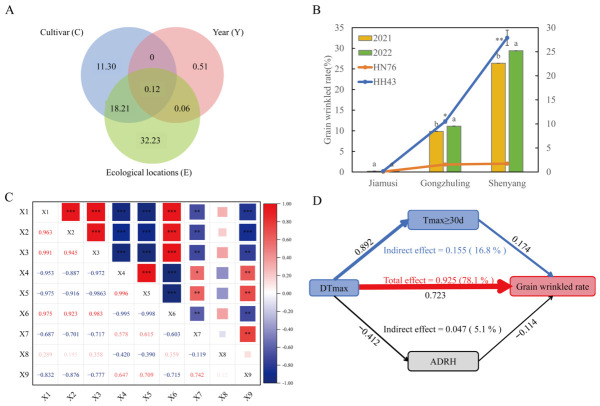
Phenotypic characterization of soybean seed wrinkling and identification of its core environmental regulatory mechanisms. (**A**): Contribution proportions of variation sources to seed-wrinkling proportion in soybean; (**B**): triple interactive effects of cultivar, ecological site and growing year on seed-wrinkling proportion of heat-sensitive cultivar HH43; Different letters represent significant differences (*p* < 0.05) among ecological locations or years within the same cultivar; * *p* ≤ 0.05, ** *p* ≤ 0.01. (**C**): correlation analysis between seed-wrinkling proportion and multiple meteorological parameters. X1, grain wrinkled rate; X2, mean daily minimum temperature (DTmin); X3, mean daily maximum temperature (DTmax); X4, accumulated precipitation (ADEP); X5, mean daily relative humidity (ADRH); X6, number of days with daily maximum temperature ≥ 30 °C (Tmax ≥ 30 d); X7, mean sunshine duration (ADSD); X8, mean wind speed (ADWV); X9, mean diurnal temperature range (ADTR). Different lowercase letters denote significant differences (*p* < 0.05) across ecological sites or cropping years for an identical cultivar. Significance levels: * *p* ≤ 0.05, ** *p* ≤ 0.01, *** *p* ≤ 0.001; (**D**): path analysis linking seed-wrinkling proportion to meteorological variables during the seed-filling stage.

Two-year multi-site variance decomposition and contribution-rate analyses were performed at three experimental locations to disentangle the individual and combined influences of cultivar genotype (C), ecological surroundings (E), and cropping year (Y) on the seed-shriveling proportion of soybean (Figure 1A, Table S1). All three main factors and their pairwise interactions exhibited statistically significant effects on seed wrinkling. Ecological conditions served as the primary determinant of phenotypic variation (F = 2546.82, *p* < 0.001), accounting for more than 50% of total variation, with genotypic divergence among cultivars ranking second (F = 2986.45, *p* < 0.001, ~30% contribution). Despite a statistically meaningful effect of growing year on seed wrinkling, its relative contribution remained negligible (0.69%), which suggests that weak year-to-year meteorological fluctuations impose limited disturbance on seed-wrinkling performance.

The interaction between cultivar genotype and ecological zone showed extremely significant statistical differences (F = 922.35, *p* < 0.001), contributing 18.33% to the total seed-wrinkling variation. The seed-wrinkling proportion of heat-susceptible cultivar HH43 rose drastically from 0.20% at the Jiamusi site to 27.90% in Shenyang, whereas heat-tolerant HN76 sustained consistently low wrinkling percentages across all experimental locations. The enlarged phenotypic divergence between the two genotypes under divergent environmental conditions confirmed that seed wrinkling is jointly driven by genetic vulnerability and site-specific abiotic stressors. The interactive effect of ecological zone × growing year reached statistical significance but contributed minimally to variation (0.18%). No obvious temperature disparities at the pre-pod-formation stage were observed among test sites, yet ambient temperatures during the seed-filling period were substantially higher in Shenyang relative to Gongzhuling and Jiamusi. A pronounced location-by-cultivar interaction specifically occurring at the seed-filling stage (F_1,12_ = 23.02, *p* = 0.003) further pinpointed this growth period as the decisive developmental stage determining seed-wrinkling susceptibility (Table S2, Figure 1B).

Sequential analyses including Pearson correlation, partial correlation, multiple regression and path modeling were implemented to screen key meteorological drivers responsible for seed-wrinkling susceptibility in heat-sensitive cultivar HH43. Correlation outputs demonstrated that temperature-related parameters across the seed-filling period served as the primary abiotic stress determinants. The average daily maximum temperature (DTmax, r = 0.991, *p* < 0.001) and cumulative days with maximum daily temperature exceeding 30 °C (Tmax ≥ 30 d, r = 0.975, *p* < 0.001) exhibited highly robust positive associations with seed-wrinkling proportion, retaining stable independent impacts when removing interfering climatic variables such as relative humidity and rainfall. While relative humidity and precipitation displayed significant negative correlations with seed shriveling, their regulatory contributions remained trivial (Figure 1C, Table S3).

Stepwise multiple regression demonstrated that nine climatic indicators jointly accounted for 95.7% of the total variation in seed-wrinkling proportion. The best-fitting predictive model retained solely DTmax and Tmax ≥ 30 d, achieving an adjusted coefficient of determination (adjusted *R*^2^) of 0.955. DTmax acted as the leading predictive variable, elevating seed-wrinkling proportion by 2.15% with each 1 °C increment, whereas Tmax ≥ 30 d functioned as a synergistic driver that increased seed wrinkling by 1.32% per additional hot day; all remaining meteorological indices provided no further predictive capacity (Tables S4 and S5). Path modeling indicated that DTmax imposed a total standardized effect of 0.925 on seed-wrinkling proportion, which was mainly derived from its direct impact (path coefficient = 0.723, 78.1% contribution) and secondarily from indirect influences mediated by Tmax ≥ 30 d (16.8% contribution). Relative humidity exhibited a negligible and statistically non-significant indirect effect, which confirms that it exerts no independent regulatory role in modulating seed wrinkling (Figure 1D).

### 2.2. Physiological Analysis—Verification of Abnormal Source–Flow–Sink Functions

#### 2.2.1. Changes in Photosynthetic Function at the Source Organ

**Figure 2:**
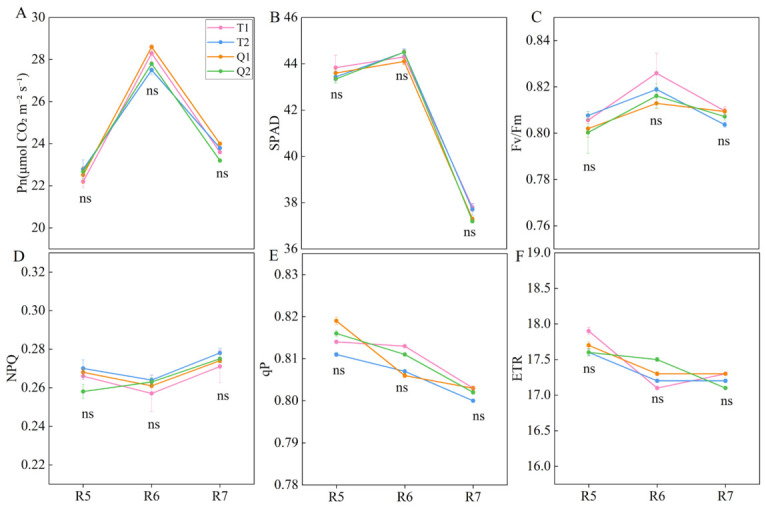
Source–leaf physiological performance of soybean grown under two contrasting ecological environments. (**A**): Leaf net photosynthetic rate; (**B**): leaf relative chlorophyll concentration; (**C**): leaf soluble sugar concentration; (**D**): leaf starch concentration; (**E**): photochemical quenching coefficient (qP); (**F**): electron transport rate (ETR). T1, heat-tolerant cultivar HN76 grown in Shenyang; T2, heat-susceptible cultivar HH43 grown in Shenyang; Q1, heat-tolerant cultivar HN76 grown in Jiamusi; Q2, heat-susceptible cultivar HH43 grown in Jiamusi.

To disentangle the relationship between seed-wrinkling susceptibility and source–leaf photosynthetic capacity under divergent ecological conditions, six core photosynthetic traits, namely, net photosynthetic rate (Pn), SPAD readings, maximum photochemical efficiency of PSII (Fv/Fm), non-photochemical quenching (NPQ), photochemical quenching coefficient (qP), and electron transport rate (ETR), were quantified at the R5, R6 and R7 seed-filling stages for both soybean genotypes across two experimental sites (Figure 2). None of the six physiological indicators exhibited significant spatial variations between Shenyang and Jiamusi at any monitored developmental stage. At the early seed-filling stage (R5), leaf function was still relatively stable, and high temperature and genotypic difference had not yet induced significant changes in photochemical quenching capacity. As plant senescence intensified at later stages, obvious differences in qP gradually appeared among treatments. These results confirmed that heat stress occurring during seed filling imposes no severe injury to leaf photosynthetic machinery, carbon-fixing ability or photosynthetic electron transport efficiency. Consequently, steady source–leaf photosynthetic performance is not the major restrictive factor responsible for seed wrinkling formation in soybean plants.

#### 2.2.2. Analysis of Sink Function Changes

**Figure 3:**
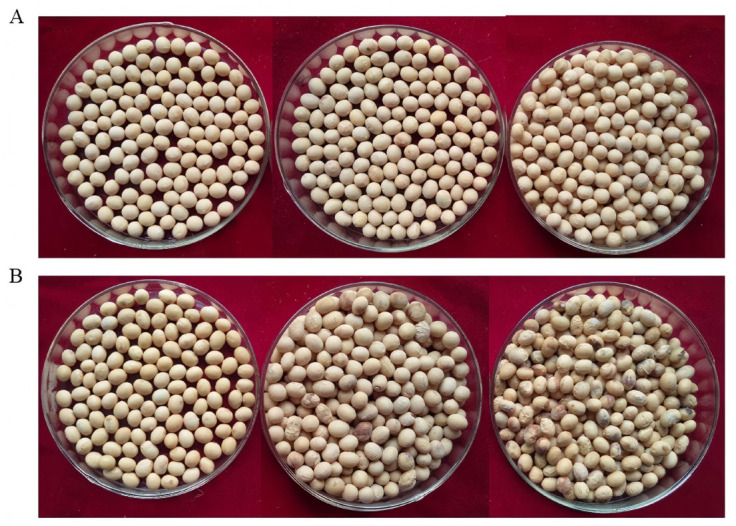
Seed phenotypes of two cultivars in different ecological locations. (**A**): Wrinkle-resistant cultivar HN76 (Left: Jiamusi, Middle: Gongzhuling, Right: Shenyang); (**B**): wrinkle-susceptible cultivar HH43 (Left: Jiamusi, Middle: Gongzhuling, Right: Shenyang). Uniform 90 mm Petri plates were adopted, with 150–200 representative seeds placed in each dish solely for phenotypic demonstration rather than yield assessment.

To further assess how diverse ecological conditions modulate the sink organ performance of soybean during the seed-filling stage, we dynamically monitored grain dry weight, grain-filling rate, starch concentration, and seed-wrinkling proportion across experimental treatments. Phenotypic analysis demonstrated that the heat-susceptible cultivar HH43 grown in Shenyang (T2) exhibited severe seed-filling defects and obvious seed wrinkling (Figure 3B). By contrast, the heat-tolerant genotype HN76 developed normally with no wrinkling under both Shenyang (T1) and Jiamusi (Q1) conditions (Figure 3A).

**Figure 4:**
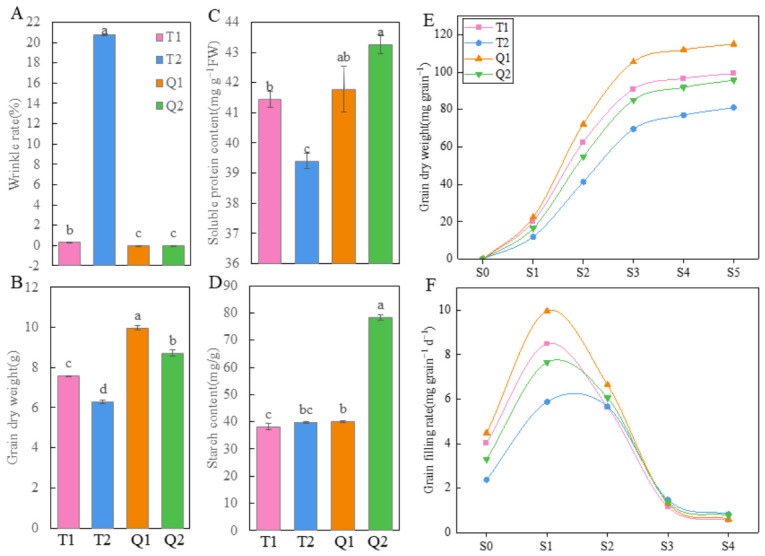
Sink organ physiological performance of soybean under two distinct ecological environments. (**A**): Wrinkled grain rate per plant; (**B**): grain dry weight at the final seed-filling stage (R7); (**C**): soluble protein content; (**D**): grain starch content; (**E**): grain-filling dynamics; (**F**): grain-filling rate. S0–S5 represent six consecutive sampling points starting from the initial seed-filling stage (R5), with 5-day intervals (S0: day 0, S1: day 5, S2: day 10, S3: day 15, S4: day 20, S5: day 25). T1, heat-tolerant cultivar HN76 grown in Shenyang; T2, heat-susceptible cultivar HH43 grown in Shenyang; Q1, heat-tolerant cultivar HN76 grown in Jiamusi; Q2, heat-susceptible cultivar HH43 grown in Jiamusi. Different lowercase letters indicate significant differences at the *p* < 0.05 level.

Quantitative phenotypic statistics revealed that the seed-wrinkling proportion of individual HH43 plants exposed to high-temperature stress in Shenyang (T2) peaked at 20.74% (Figure 4A). Both soybean genotypes exhibited a typical sigmoidal increase in seed dry mass from the early seed-filling stage (S0) to physiological maturity (S6). The heat-tolerant cultivar HN76 retained consistent seed-filling dynamics across ecological sites, with its maximum filling rate observed at 15 days after the initiation of seed filling (S3) and negligible spatial variation between locations. Conversely, heat-vulnerable HH43 under Shenyang conditions (T2) showed postponed seed-filling commencement, sharply diminished peak filling capacity, truncated filling duration and depressed dry matter accumulation. These adverse phenotypic changes substantially reduced single-seed dry mass and 100-seed weight relative to HH43 plants cultivated in Jiamusi (Q2). Overall, constrained seed-filling capacity that causes defective seed development serves as the core sink-related characteristic responsible for soybean seed wrinkling under heat stress.

In parallel, HH43 seeds cultivated under high-temperature conditions in Shenyang exhibited markedly reduced starch and soluble protein abundances, which coincided with an increased seed-wrinkling proportion and further validated the suppression of sink tissue functionality by heat stress. Integratively, high-temperature exposure debilitated seed sink strength and restricted dry matter accumulation in HH43, which constituted the dominant physiological factor triggering seed wrinkling defects. Relative to HH43 plants grown in Jiamusi (Q2), the Shenyang-cultivated counterparts (T2) displayed pronounced reductions in soluble protein level, starch concentration, seed dry mass, and seed-filling efficiency (Figure 4B–F).

#### 2.2.3. Analysis of Flow Function Changes

To clarify the association between seed-wrinkling formation and assimilate translocation efficiency (transport capacity) under divergent ecological conditions during the seed-filling period, we systematically determined sucrose concentration gradients throughout the leaf–stem–husk–seed transport pathway, key activities of sucrose-metabolizing enzymes in pod husks and developing seeds, pod husk ATP accumulation and ATPase activity, and seed AGPase activity across the R5, R6, and R7 developmental stages. This multi-index physiological assay aimed to uncover the central mechanism underlying heat-suppressed assimilate translocation in heat-stressed soybean plants.

**Figure 5:**
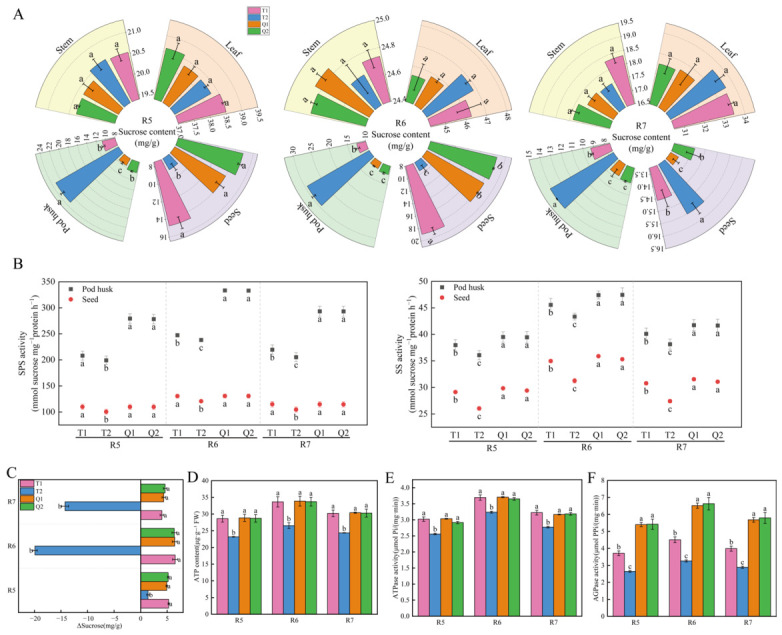
Assimilate transport performance of soybean under two contrasting ecological environments. (**A**): Sucrose content in stem, leaf, pod husk and seed at R5, R6 and R7; (**B**): sucrose phosphate synthase (SPS) and sucrose synthase (SS, cleavage direction) activities in pod husk and seed at R5, R6 and R7; (**C**): sucrose concentration difference (ΔSucrose) between pod husk and seed at R5, R6 and R7; (**D**): ATP content in pod husk at R5, R6 and R7; (**E**): ATPase activity in pod husk at R5, R6 and R7; (**F**): AGPase activity in seed at R5, R6 and R7. T1, HN76 planted in Shenyang; T2, HH43 planted in Shenyang; Q1, HN76 planted in Jiamusi; Q2, HH43 planted in Jiamusi. Different lowercase letters indicate significant differences at the *p* < 0.05 level.

Sucrose concentrations in leaves and stems exhibited no obvious spatial variations throughout the three key seed-filling stages across experimental sites (Figure 5A). This finding verified that source tissues maintained steady photoassimilate synthesis and intact long-distance carbon translocation from leaves to pod husks, which ensured adequate upstream carbon provision and unimpeded mass flow. With the advancement of seed development, pod husk sucrose levels in Jiamusi plants stayed consistently low, and a positive sucrose gradient between pod husks and seeds (ΔSucrose) guaranteed uninterrupted assimilate allocation to developing seeds. Conversely, heat-vulnerable HH43 cultivated in Shenyang displayed progressive sucrose accumulation in pod husks beginning at the R5 stage, with a substantially higher peak at the R6 stage relative to Jiamusi-grown plants. The corresponding ΔSucrose eventually turned negative (Figure 5C), demonstrating pronounced carbon retention and persistent transport restriction within pod husk tissues under high-temperature stress.

Stage-dependent quantification of key sucrose-metabolizing enzymes revealed that the cleavage activity of sucrose synthase (SS) in pod husks of heat-susceptible HH43 grown in Shenyang was consistently and significantly reduced from R5 to R7 relative to Jiamusi-cultivated plants, which substantially impaired the sucrose unloading capability of pod tissues (Figure 5B). In parallel, pod husk ATP accumulation, ATPase activity, and seed AGPase activity exhibited persistent and significant reductions across the entire seed-filling period (R5–R7), with the most dramatic inhibitory effects observed at the R6 stage (Figure 5D–F). These physiological responses indicated that high-temperature stress continuously repressed energy supply and carbohydrate activation in pod husks, thereby diminishing the driving force required for sucrose transmembrane transport. Integratively, this sequential phenotypic and biochemical evidence illustrates that heat stress breaks the intrinsic coupling between assimilate flow and energy metabolism in pod husks, restricts the translocation of photoassimilates to developing seeds, and ultimately constitutes the core physiological pathway responsible for heat-induced seed wrinkling in soybean.

### 2.3. Transcriptomic Analysis

#### 2.3.1. Detection of Differentially Expressed Genes

**Figure 6:**
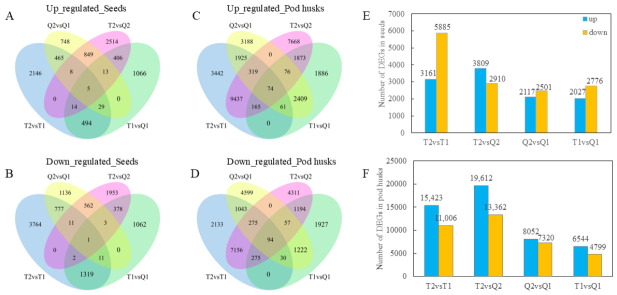
Differentially expressed genes in pod husks and seeds between the two ecological locations. (**A**): Venn plots of up-regulated genes in seeds; (**B**): Venn plots of down-regulated genes in seeds; (**C**): Venn plots of up-regulated genes in pod husks; (**D**): Venn plots of down-regulated genes in pod husks; (**E**): number of differentially expressed genes in seeds; (**F**): number of differentially expressed genes in pod husks.

To further elucidate the molecular mechanisms governing the differential responses of the seed source–sink-flow relationship to varying ecological locations, whole-genome transcriptome sequencing was conducted on pod husks and seeds of HH43 and HN76 grown in two distinct ecological sites. To assess the reliability of transcriptome data and sample grouping, PCA was performed on seed coat and seed transcriptome samples (Figure S3). In both tissues, biological replicates from the same group were tightly clustered, which indicates high reproducibility. Meanwhile, samples from different ecological conditions (T1/T2/Q1/Q2) were clearly separated along the first two principal components, which reflects distinct transcriptomic profiles under different environments. These results confirmed the good quality of the sequencing data and the validity of the subsequent analyses. Venn diagram results showed the following for the comparisons of T2 vs. T1, Q2 vs. Q1, T2 vs. Q2, and T1 vs. Q1: in seeds, 3161, 3809, 2117, and 2027 genes were up-regulated, respectively, and 5885, 2910, 2501, and 2776 genes were down-regulated, respectively; and in pod husks, 15,423, 19,612, 8052, and 6544 genes were up-regulated, respectively, and 11,006, 13,362, 7320, and 4799 genes were down-regulated, respectively (Figure 6A−F). Notably, in the seed comparison groups of T2 vs. T1 and Q2 vs. Q1, 507 genes were co-up-regulated and 800 genes were co-down-regulated; in the T2 vs. Q2 and T1 vs. Q1 comparison groups, 438 genes were co-up-regulated and 384 genes were co-down-regulated (Figure 6A,B). In the pod husk comparison groups of T2 vs. T1 and Q2 vs. Q1, 2379 genes were co-up-regulated; in the T2 vs. Q2 and T1 vs. Q1 comparison groups, 2188 genes were co-up-regulated (Figure 6C,D). This indicates that the changes in gene expression caused by differences between ecological locations were significantly greater than those between cultivars, and pod husks were more sensitive to environmental responses than seeds. Compared to the T1 vs. Q1 comparison group, the seed T2 vs. Q2 comparison group contained 1782 more up-regulated genes and 134 more down-regulated genes; by contrast, the pod husk T2 vs. Q2 comparison group had 13,068 additional up-regulated genes and 8563 more down-regulated genes. HH43 exhibited more DEGs than HN76 (Figure 6A−F), which indicates that HH43 was sensitive to the Shenyang ecological locations, especially in pod husks, and responded to the stress.

#### 2.3.2. Functional Classification of Differentially Expressed Genes

**Figure 7:**
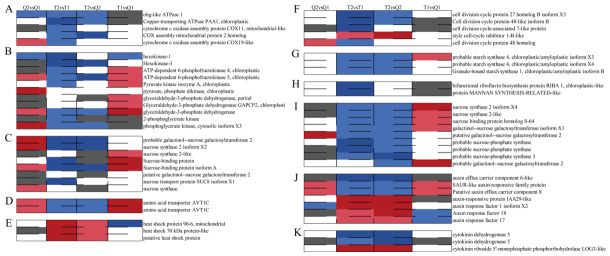
PageMan display of coordinated changes in selected gene categories activated by different ecological environments. (**A**): Mitochondrial electron transport in pod husks; (**B**): glycolysis in pod husks; (**C**): sucrose transport stress in pod husks; (**D**): amino acid transport in pod husks; (**E**): heat stress in pod husks; (**F**): cell proliferation cycle in seeds; (**G**): grain starch synthesis; (**H**): grain protein synthesis; (**I**): grain sucrose degradation; (**J**): grain auxin response; (**K**): grain cytokinin synthesis. (Dark blue, *p* ≤ 0.001, significantly down; light blue, 0.001< *p* ≤ 0.05, down-regulated; deep red, *p* ≤ 0.001, significantly up-regulated; light red, 0.001 < *p* ≤ 0.05, up-regulated; grey, *p* > 0.05, not significant).

To explore the relationship among enriched transcripts of the wrinkle-susceptible cultivar under different ecological locations, PageMan (v1.9.1, Max Planck Institute of Molecular Plant Physiology, Potsdam, Germany) analysis was used to generate clusters for all annotated differentially expressed genes. This study focused on analyzing differentially expressed genes belonging to categories involving pod husk mitochondrial electron transport, stress, glycolysis, sucrose transport, amino acid transport, the seed cell proliferation cycle, starch synthesis, protein synthesis, sucrose decomposition, auxin response, and cytokinin synthesis (Figure 7). In pod husks, DEGs related to ATP synthase, glycolysis, sucrose transport, and amino acid transport were significantly down-regulated and enriched in both the T2 vs. T1 and T2 vs. Q2 comparison groups (Figure 7A−D); heat-shock transcription factor-related DEGs exhibited significant up-regulation and enrichment in the two comparison groups of T2 vs. T1 and T2 vs. Q2 (Figure 7E). In seeds, DEGs related to cell cycle promotion were significantly down-regulated and enriched in both the T2 vs. T1 and T2 vs. Q2 comparison groups, while DEGs related to cell cycle inhibition were significantly up-regulated and showed enrichment in both the T2 vs. T1 and T2 vs. Q2 comparison groups (Figure 7F); significant downregulation and enrichment of DEGs related to starch synthesis, protein synthesis, and sucrose decomposition were observed in the T2 vs. T1 comparison group (Figure 7G−I); DEGs related to auxin promotion response were significantly down-regulated and enriched in both the T2 vs. T1 and T2 vs. Q2 comparison groups, while DEGs related to auxin inhibition response were significantly up-regulated and showed enrichment in both the T2 vs. T1 and T2 vs. Q2 comparison groups (Figure 7J); and both the T2 vs. T1 and T2 vs. Q2 comparison groups exhibited significant down-regulation and enrichment of DEGs involved in auxin promotion responses related to cytokinin synthesis promotion, while DEGs associated with auxin promotion responses linked to cytokinin inhibitors were significantly up-regulated and enriched in these two groups (Figure 7K). The above results revealed that short-term high temperatures at the grain-filling stage in Shenyang down-regulated the expression of sucrose transporter genes and energy metabolism genes in the pod husks of HH43 and down-regulated the expression of starch synthesis genes in the seeds, resulting in the insufficient supply and regulatory disorders of the substances and energy required for seed development, thereby triggering seed wrinkling.

### 2.4. Construction and Analysis of Weighted Gene Co-Expression Network

To unravel the regulatory association between the physiological phenotypes and transcriptomic profiles of soybean under distinct ecological habitats, we implemented weighted gene co-expression network analysis (WGCNA) for systematic molecular screening.

**Figure 8:**
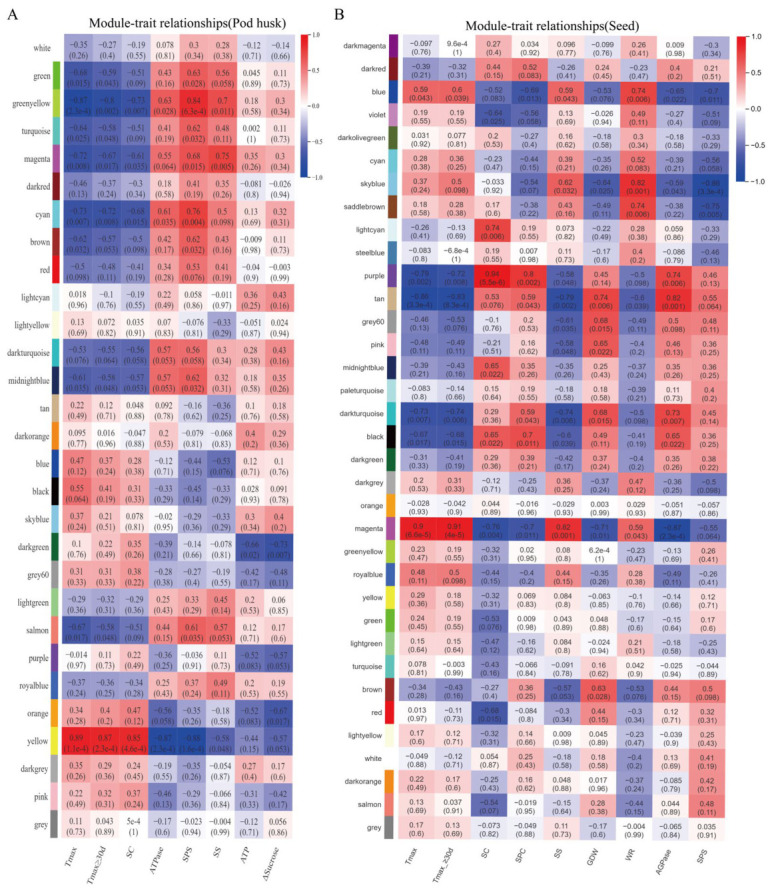
Module–trait correlation heatmap of gene co-expression modules in pod husks and seed. (**A**): Rows represent co-expression modules; columns represent temperature indices (Tmax, Tmax ≥ 30 d), pod husk sucrose content (SC), ATPase activity, sucrose phosphate synthase (SPS) activity, sucrose synthase (SS) activity, ATP content, and sucrose concentration difference (ΔSucrose). (**B**)**:** Rows represent co-expression modules; columns represent temperature indices (Tmax, Tmax ≥ 30 d), seed sucrose content (SC), soluble protein content (SPC), sucrose synthase (SS) activity, grain dry weight (GDW), wrinkling rate (WR), AGPase activity, and sucrose phosphate synthase (SPS) activity. Red color denotes significant positive correlations, whereas blue color represents significant negative correlations.

In pod husk tissues (Figure 8A), the yellow module was screened out as the pivotal heat-responsive regulatory module. This module showed significant positive associations with high-temperature meteorological indicators and pod husk sucrose levels while displaying negative correlations with ATPase activity and the activities of key sucrose-metabolizing enzymes. These transcriptomic associations demonstrated that heat stress activates stress-adaptive responses and simultaneously inhibits energy metabolism and sucrose translocation capacity, thereby inducing excessive sucrose accumulation in pod husks. By contrast, the green module, which governs normal sucrose metabolic processes, was markedly repressed under high-temperature conditions, which further constrained assimilate translocation efficiency in heat-stressed soybean plants.

In seed tissues (Figure 8B), the magenta module was defined as the core heat-responsive regulatory module. It displayed positive correlations with thermal environmental factors and seed-wrinkling proportion while showing negative associations with seed sucrose concentration, soluble protein level and AGPase activity, which indicates a heat-triggered attenuation of seed sink strength. The purple module, which maintains normal seed-filling progression, was significantly repressed under heat stress. Integratively, our findings uncovered a dual-layered regulatory cascade: high-temperature stress disrupts sucrose translocation in pod husks and weakens seed sink capacity, eventually causing seed-shriveling defects in soybean.

**Figure 9:**
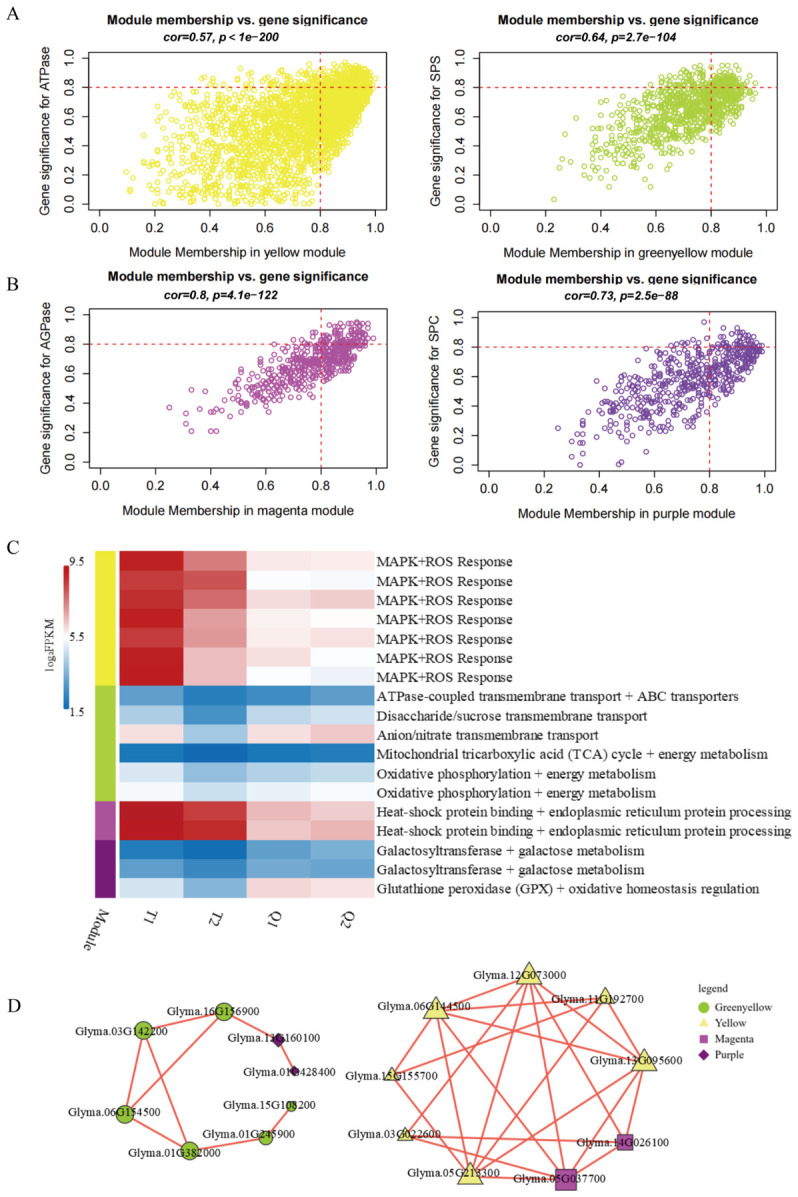
Co-expression network analysis of grain wrinkling-related modules identified by WGCNA. (**A**): Scatter plot of gene significance (GS) vs. module membership (MM) in the yellow and green-yellow module of pod husks; (**B**): scatter plot of gene significance (GS) vs. module membership (MM) in the magenta and purple module of seeds; (**C**): expression heatmap of core hub genes under different treatments. These hub genes were identified from WGCNA modules and jointly enriched by GO and KEGG analyses. Gene expression levels were normalized by log_2_-transformed FPKM. The red–blue gradient represents up- and down-regulated expression, and genes are clustered according to their corresponding modules and biological functions. (**D**): Co-expression network constructed using hub genes with dual enrichment of GO and KEGG from the four core modules.

GS-MM correlation analysis was further performed on four key co-expression modules: the yellow and green-yellow modules derived from pod husks, and the magenta and purple modules from developing seeds. In pod husk tissues, genes assigned to the yellow module exhibited significant negative correlations with ATPase activity, which diminished the energy-driven motive force required for sucrose translocation. In comparison, genes in the green-yellow module displayed marked positive associations with SPS activity, supporting intact sucrose metabolic pathways (Figure 9A). Within seed tissues, magenta-module genes were tightly negatively correlated with AGPase activity and, thus, repressed starch biosynthesis; by contrast, purple-module genes were positively linked to seed sucrose concentration to preserve normal seed-filling capacity (Figure 9B). These molecular results confirm that heat stress establishes a dual regulatory mode: stress-induced responses and suppressed carbohydrate metabolism in pod husks, coupled with activated stress signaling and impaired seed-filling progression in seeds. This cascade ultimately restricts sucrose translocation and weakens seed sink capacity under high-temperature conditions.

Hub genes within the yellow module of pod husks were significantly enriched in the MAPK signaling cascade, phytohormone signal transduction, oxidative stress responses, and oxidative phosphorylation pathways (Figure S1A, Table S6). These functional enrichments suggest that heat stress initiates oxidative stress via upstream signal perception, represses ATP biosynthesis, weakens the energy-driven motive force for sucrose translocation, induces sucrose accumulation in pod husks, and accelerates pod husk senescence. The green-yellow module was enriched in carbohydrate metabolism, energy provision, and assimilate transport pathways, which collectively maintain an intact sucrose translocation capacity in pod husks (Figure S1B, Table S6). This module was markedly repressed under high-temperature stress and cooperated synergistically with the yellow module to block assimilate translocation from pod husks to seeds.

In developing seeds, hub genes of the magenta module were significantly enriched in heat-shock protein functions, endoplasmic reticulum protein processing, glycolysis, and ubiquitination-mediated degradation pathways (Figure S1C, Table S6). These core genes participate in heat-stress responses and disrupted energy metabolism, which consequently suppress starch biosynthesis and seed-filling progression. The purple module was enriched in galactose metabolism, protein folding processes, and antioxidant homeostasis, all of which support carbohydrate anabolism and intact seed-filling capacity (Figure S1D, Table S6). This module was markedly repressed under high-temperature stress and synergistically interacted with the magenta module to attenuate seed sink capacity.

To further verify the biological functions of key co-expression modules, a total of 18 hub genes with consistent enrichment in both GO terms and KEGG pathways were screened from the yellow and green-yellow modules of pod husks, as well as from the magenta and purple modules of developing seeds. The transcriptional patterns of these hub genes are visualized in a heatmap (Figure 9C). Heat stress significantly up-regulated the expression levels of stress-responsive genes in both pod husks and seeds, whereas it suppressed genes involved in sucrose translocation and seed-filling metabolism. Compared to the heat-susceptible cultivar HH43, the heat-tolerant genotype HN76 displayed relatively mild transcriptional alterations, which were highly consistent with the differential physiological phenotypes observed between the two cultivars. Based on these core hub genes, a refined gene co-expression network was subsequently constructed (Figure 9D). Stress-related and metabolism-associated genes formed independent co-expression clusters exhibiting antagonistic regulatory relationships. Collectively, this transcriptomic evidence further demonstrated that high-temperature stress disrupts the holistic source–flow–sink balance of soybean via a dual regulatory strategy involving activated stress signaling and repressed carbohydrate metabolism.

### 2.5. qRT-PCR Validation of Key Hub Genes

**Figure 10:**
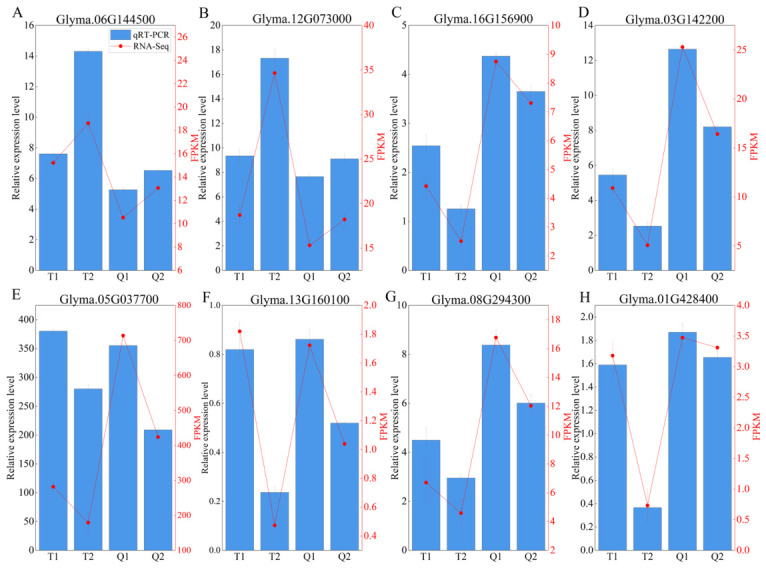
Validation of key hub gene expression by quantitative real-time PCR. (**A**,**B**): Hub genes of the green module; (**C**,**D**): hub genes of the green-yellow module; (**E**): hub genes of the magenta module; (**F**–**H**): hub genes of the purple module.

To validate the reproducibility of the transcriptomic datasets, eight representative hub genes screened from the four key co-expression modules were subjected to quantitative real-time PCR (qPCR) verification. The expression profiles of these candidate genes across different ecological environments and soybean cultivars were highly consistent with the RNA-seq results. Under high-temperature conditions (T1, T2), stress-responsive genes belonging to the yellow module of pod husks (Figure 10A,B) and the magenta module of developing seeds (Figure 10E) were significantly up-regulated. In parallel, sucrose translocation-associated genes in the green-yellow module of pod husks (Figure 10C,D), together with genes responsible for carbohydrate biosynthesis and sink capacity maintenance in the seed purple module (Figure 10F–H), were substantially repressed.

Comparative analysis between cultivars showed that the heat-tolerant genotype HN76 (T1) exhibited a substantially attenuated induction of stress-related genes and a milder repression of metabolic genes under heat stress compared to the heat-susceptible cultivar HH43 (T2). This distinct transcriptional variation confirmed the stronger thermotolerance of HN76. The high consistency of gene expression trends between the qPCR quantification and RNA-seq data validated the reliability of the identified hub genes, providing solid molecular evidence illustrating how heat stress disrupts the source–flow–sink balance and ultimately triggers seed wrinkling in soybean.

## 3. Discussion

### 3.1. Cultivar and Environment Interaction Dominates the Formation of Soybean Seed Wrinkling

Crop phenotypic variation is the result of the synergistic effect of genotype and environment, and clarifying their interaction effect is a prerequisite for analyzing the mechanism of stress resistance differences [32]. This study confirmed through multivariate analysis of variance that the cultivar × environment interaction effect is the primary factor driving the variation in soybean seed-wrinkling rate. The environmental differences among ecological locations and the genetic differences among cultivars are the first and second key main effect factors, respectively, and the grain-filling stage is the critical growth period affecting seed wrinkling. Consistent with the conclusions drawn by Osman [18] and Fan [19] in previous studies, our result confirms that “genotypic differences in the response of soybean seed development to environmental stress are significant, and the grain-filling stage acts as a sensitive window period.” In this study, a cross-latitude synchronous experiment was conducted to simplify the differences between ecological locations into a single variable of temperature gradient. The data demonstrated that during the seed-filling stage, Tmax in Shenyang exceeded that in Jiamusi by 3.8 °C, which was extremely significantly positively correlated with the number of wrinkled seeds per plant (r = 0.991, *p* < 0.001, Figure 1). Similar to Li’s [33] research on maize subjected to heat stress during the grain-filling stage—where 42 °C/30 °C heat stress caused a sharp drop in the grain dry matter accumulation rate and significantly reduced the 1000-grain weight, grain-filling rate, and seed setting rate—our finding suggests that temperature variations in natural fields can induce inadequate seed development or even seed abortion. On an ecological scale, this discovery confirmed that seed-filling disorders were not merely inherent genotypic traits but rather the result of the interaction between high-temperature-sensitive genotypes and persistent high temperatures. As shown in Figure 10, wrinkled seeds of the heat-sensitive genotype also presented obvious discoloration besides morphological defects. Seed color is closely linked to the accumulation of pigments and phenolic compounds, whose metabolism is highly vulnerable to heat stress during seed filling [34]. Because the current study mainly focused on seed wrinkling caused by disrupted carbon and energy metabolism, we did not quantify these secondary metabolites. We speculate that the heat-induced disorder of primary metabolism further disturbs secondary metabolic processes in seeds, jointly leading to morphological and color abnormalities. Related quantitative detection will be carried out in our future research to clarify the underlying mechanism.

### 3.2. Abnormal Pod Husk Transport Function and Energy Metabolism Were the Core Physiological Mechanisms of Seed Wrinkling

The coordinated operation of the “source-sink-flow” system is the basis of soybean seed filling, and the transport efficiency of the “flow” directly determines the supply rate of photosynthates to seeds [35,36]. The physiological analysis in this study showed that there were no significant differences in source strength (leaf photosynthetic rate, chlorophyll content, etc.) between the two cultivars grown in the three locations, while the differences were mainly reflected in the functions of “flow” (pod husks) and “sink” (seeds): HH43 grown in Shenyang had significantly decreased pod husk sucrose transport capacity and insufficient ATP synthesis, while the seed starch synthesis efficiency was reduced. This result negated the traditional cognition that “insufficient source strength caused seed wrinkling”, clarified that the functional disorder of “flow” organs (pod husks) was the core limiting factor, and confirmed the initial hypothesis of this study. As a key channel for the transport of assimilates from stems to seeds, the sucrose transport capacity and energy supply of pod husks were directly related to transport efficiency [37,38]. In this study, short-term high temperatures in Shenyang inhibited the activity of sucrose transporters in the pod husks of HH43 and reduced ATP synthesis at the same time—insufficient energy further inhibited the transmembrane transport function of sucrose transporters, forming a vicious cycle of “decreased transport capacity-insufficient energy”, leading to the retention of assimilates in pod husks and failure to supply seeds in a timely manner. Due to the lack of sufficient carbohydrate substrates, the activity of key starch synthesis enzymes in seeds decreased, ultimately leading to insufficient filling and seed wrinkling. By contrast, HN76 ensured the supply of assimilates to seeds by maintaining stable sucrose transport capacity and energy synthesis efficiency in pod husks, which was consistent with its phenotype of normal seed development. This finding revealed a new mechanism of “flow-energy coupling” regulating soybean seed development and enriched the physiological theory of soybean response to abiotic stress.

### 3.3. Transcriptomic Analysis of the Molecular Regulatory Network of Seed Wrinkling

Transcriptomic analysis provides key support for elucidating the molecular mechanisms underlying differences in physiological functions [39,40,41,42]. The transcriptomic results of this study showed that in the pod husks of HH43 grown in Shenyang, DEGs associated with ATP synthase, glycolysis (a key pathway of energy metabolism), sucrose transport and amino acid transport were significantly down-regulated and enriched. By contrast, DEGs related to heat-shock transcription factors were markedly up-regulated and enriched. This result confirmed the changes in physiological indicators at the molecular level: the down-regulation of energy metabolism-related genes led to reduced ATP synthesis, the down-regulation of sucrose transport genes inhibited transport function, and the up-regulation of heat-shock transcription factors was a stress response of plants to high-temperature stress, but it failed to effectively alleviate functional damage [43,44,45]. In seeds, DEGs related to cell cycle promotion were down-regulated and those related to cell cycle inhibition were up-regulated in HH43 grown in Shenyang; meanwhile, DEGs related to starch synthesis, protein synthesis, and sucrose decomposition were significantly down-regulated. The changes in the expression of cell cycle regulatory genes indicated that high-temperature stress inhibited the division and elongation of seed cells, leading to insufficient sink capacity construction [46,47]; the down-regulation of substance synthesis-related genes directly reduced the efficiency of dry matter accumulation, and the dual effects exacerbated seed wrinkling. When HN76 was grown in Shenyang, there were no significant abnormalities in the expression of the above key genes, which was consistent with its stable physiological functions and phenotype. This result constructed a molecular regulatory chain of “high-temperature stress-down-regulation of pod husk energy and transport genes-down-regulation of seed cell cycle and synthesis genes-seed wrinkling”, clarifying the molecular basis of inter-cultivar differences.

### 3.4. Mining and Validation of Core Regulatory Modules and Hub Genes

Weighted gene co-expression network analysis (WGCNA) has been widely recognized as a powerful tool for identifying pivotal regulatory genes responsible for complex agronomic traits in crops [48,49]. Integrating WGCNA with GS-MM correlation analysis and functional enrichment annotation, the present study successfully distinguished heat-responsive co-expression modules specifically operating in pod husks and seeds, thereby establishing a two-tiered molecular regulatory cascade that modulates heat-affected seed development in soybean. The yellow module in pod husks was validated as the central heat-responsive module, which was enriched in genes associated with MAPK signaling, phytohormone transduction, oxidative stress defense, and oxidative phosphorylation pathways. Under high-temperature stress, excessive activation of upstream signaling cascades induced intensive oxidative stress in pod husk tissues, inhibited ATP biosynthesis, diminished the energy-driven driving force for sucrose translocation, and ultimately accelerated pod husk senescence. In comparison, the green-yellow module, which dominates carbohydrate metabolism and energy-dependent assimilate translocation, was substantially repressed by heat stress. The repressed metabolic module cooperated synergistically with the activated stress module to severely impede carbon export from pod husks, thereby elucidating the molecular basis of heat-induced sucrose retention in pod husk tissues.

In developing seeds, the magenta module was enriched in genes associated with heat-shock protein metabolism and glycolysis pathways, which collectively mediate heat-stress adaptation and energy dysregulation, ultimately suppressing starch biosynthesis. By contrast, the purple module sustains antioxidant homeostasis and carbohydrate anabolism to support normal seed-filling progression; however, heat-induced repression of this module directly impairs seed sink capacity [50]. Subsequent GS-MM correlation analysis further verified that the hub genes derived from these functional modules were tightly correlated with core enzymatic activities governing sucrose metabolism and energy supply. Stress-responsive and metabolic genes formed antagonistic co-expression clusters, which demonstrates that heat stress disrupts pod husk assimilate translocation and seed sink functionality through a dual regulatory strategy: the activation of stress signaling pathways and the simultaneous repression of metabolic pathways. Such transcriptional reprogramming ultimately breaks the holistic source–flow–sink balance in heat-stressed soybean plants. The high concordance between the qPCR validation results and transcriptomic profiles confirmed the reliability of these core hub genes, which can serve as promising candidate genes for molecular breeding to improve soybean thermotolerance. Collectively, the 18 hub genes identified in this study form a central regulatory network that modulates source–flow–sink coordination during seed filling under high-temperature conditions. Transcriptional variations in these key genes adequately explain the integrated physiological mechanism by which heat stress induces sucrose accumulation in pod husks, restricts assimilate translocation toward developing seeds, and ultimately triggers defective seed sink performance and seed wrinkling in heat-susceptible soybean cultivars.

## 4. Materials and Methods

### 4.1. Plant Materials and Experimental Design

From 2021 to 2022, field trials were performed at three experimental sites: the research base of the Jiamusi Branch of Heilongjiang Academy of Agricultural Sciences (49°49′ N, 130°17′ E), the Gongzhuling Experimental Station of Jilin Academy of Agricultural Sciences (43°30′ N, 124°48′ E), and the test field of Shenyang Agricultural University (41°82′ N, 123°57′ E). Two soybean cultivars with contrasting seed-wrinkling traits were selected: Heihe43 (HH43, wrinkling-sensitive) and Henong76 (HN76, wrinkling-tolerant) (Figure 10). A planting density of 300,000 plants/ha was adopted, using a narrow-ridge double-row layout with 60 cm between ridges and 13 cm between seed belts on each narrow ridge. Each experimental plot comprised 6 rows, with a row length of 5 m, and all treatments were replicated 3 times. Two seeds were sown per hole, and thinning was performed to retain a single seedling per hole. Using three experimental sites with distinct climatic conditions, Jiamusi has a temperate continental monsoon climate, recording a mean annual temperature (MAT) of 3.0 °C and annual precipitation (AP) of 530 mm; Gongzhuling is characterized by a temperate semi-humid continental monsoon climate, with a MAT of 5–6 °C and an AP range of 450–600 mm; Shenyang, by contrast, features a warm temperate semi-humid continental monsoon climate, with a MAT of 8.5 °C and an AP of 750 mm. The basic soil fertility at the three experimental sites is shown in Table S7.

### 4.2. Statistics of Environmental Meteorological Factors

Meteorological data from 2021 to 2022 were collected from the Meteorological Station of the College of Agronomy, Shenyang Agricultural University, the Gongzhuling Experimental Meteorological Station of Jilin Academy of Agricultural Sciences, and the Meteorological Station of Jiamusi Branch of Heilongjiang Academy of Agricultural Sciences. Meteorological factors during three growth stages (R3, R5 and R8) of soybeans in the three experimental environments were statistically analyzed. The analyzed meteorological factors included maximum daily temperature (DTmax), minimum daily temperature (DTmin), number of days with daily maximum temperature ≥ 30 °C (Tmax ≥ 30 d), average daily temperature range (ADTR), average daily relative humidity (ADRH), average daily effective precipitation (ADEP), average daily sunshine duration (ADSD), and average daily wind velocity (ADWV) in the whole growth season.

### 4.3. Determination of Photosynthetic Parameters

Field measurements were conducted continuously from 2021 to 2022. The net photosynthetic rate (Pn) of the third fully expanded trifoliate leaf was determined using a LI-6400 portable photosynthesis system (LI-COR, Lincoln, NE, USA) during 8:00–9:00 a.m. at three critical seed-filling stages, namely, the initial (R5), peak (R6), and late (R7) stages. Leaf relative chlorophyll content (SPAD value) was measured with a SPAD-502 chlorophyll meter (Konica Minolta Sensing, Inc., Osaka, Japan). For each treatment, 15 uniformly grown soybean plants (five plants for each biological replicate) were randomly selected and dark-adapted for 30 min. Subsequently, a PAM-2500 modulated fluorometer (Heinz Walz GmbH, Effeltrich, Bavaria, Germany) was used to determine chlorophyll fluorescence parameters, including the maximum quantum yield of photosystem II (Fv/Fm), non-photochemical quenching coefficient (NPQ), photochemical quenching coefficient (qP), and electron transport rate (ETR).

### 4.4. Dynamics of Seed-Filling Progress

Sampling was performed at 5-day intervals from the initiation of seed filling until physiological maturity across the two consecutive growing seasons. For each experimental plot, pods were harvested from five randomly selected soybean plants, and pod husks and developing seeds were manually separated. All fresh samples were first subjected to heat inactivation at 105 °C for 30 min, followed by oven-drying at 80 °C to a constant weight. The dry weights of processed samples were then measured using an electronic analytical balance. The sampling time points were defined as S0 (initial seed-filling stage), S1 (5 days after the initiation of seed filling), S2 (10 days after the initiation of seed filling), S3 (15 days after the initiation of seed filling), S4 (20 days after the initiation of seed filling), and S5 (25 days after the initiation of seed filling). The seed-filling rate (mg·seed^−1^·d^−1^) was calculated as the difference in seed dry weight between two consecutive sampling time points divided by the corresponding sampling interval. Each treatment was established with three biological replicates, and seed-filling dynamic curves were generated based on the measured phenotypic data.

### 4.5. Determination of Sugar Content, Sugar-Metabolizing Enzyme Activities and Sucrose Gradient

Ten intact pods at consistent nodal positions were collected at the initial (R5), peak (R6), and late (R7) seed-filling stages and subsequently dissected to separate pod husks and developing seeds. The anthrone–sulfuric acid colorimetric method was applied to quantify soluble sugar and starch concentrations in both pod husk and seed tissues [51], while sucrose content was measured using the resorcinol colorimetric assay [52,53]. The sucrose concentration gradient between pod husks and seeds was calculated as ΔSucrose = seed sucrose content − pod husk sucrose content, which reflects the driving force and translocation efficiency of photoassimilate transport from pod husks to developing seeds. Key sucrose-metabolizing enzymes, including sucrose phosphate synthase (SPS), cleavage-direction sucrose synthase (SUS), acid invertase (AI), and neutral invertase (NI), were assayed via a spectrophotometric method [54]. Sucrose phosphate synthase (SPS) activity was determined using a colorimetric method. The reaction mixture was incubated at 37 °C for 30 min, and the absorbance was measured at 480 nm for activity calculation. ADP-glucose pyrophosphorylase (AGPase) activity was assayed via an enzyme-coupled reaction system. The production rate of NADPH was continuously monitored spectrophotometrically at 340 nm to calculate enzyme activity.

### 4.6. Pod Husk ATP Content and Activities of Energy-Metabolizing Enzymes

Pod husk samples at identical nodal positions were collected at the initial (R5), peak (R6), and late (R7) seed-filling stages. All fresh samples were immediately wrapped with aluminum foil, snap-frozen in liquid nitrogen, and stored at −80 °C for subsequent biochemical analyses. The contents of adenosine triphosphate (ATP), as well as the activities of ATPase and ADP-glucose pyrophosphorylase (AGPase), were determined using an enzyme-coupled spectrophotometric method [55]. Specifically, ATP content reflects the energy supply capacity of pod husks, ATPase activity represents the potential for energy hydrolysis and energy release, and AGPase activity characterizes carbohydrate activation and precursor synthesis for photoassimilate translocation. Collectively, these three physiological indicators comprehensively evaluate the energy coupling status that dominates photoassimilate transport efficiency in soybean pod husks.

### 4.7. RNA Extraction, Library Preparation and Sequencing

For total RNA extraction, the MiniBEST Universal RNA Extraction Kit (Takara, Kusatsu, Japan) was employed to isolate RNA separately from pod husks and seeds of the two soybean cultivars (HH43 and HN76) grown in Shenyang and Jiamusi sites at the grain-filling stage. After extraction, the RNA concentration was quantified using a NanoDrop 2000C ultramicro spectrophotometer (Waltham, MA, USA). For RNA integrity assessment, 3 μL of RNA solution was subjected to 1% agarose gel electrophoresis at a constant voltage of 180 V for 10 min, which enabled visualization of RNA bands to verify integrity. Subsequently, cDNA synthesis and library preparation were performed as follows: Single-stranded cDNA was first synthesized using Takara’s PrimeScript™ RT Reagent Kit (Perfect Real Time, Kusatsu, Japan). Next, DNA polymerase I and RNase H were employed to synthesize double-stranded cDNA while stripping away the mRNA template. DNA fragment 3′ ends were adenylated, and hairpin-loop NEBNext adapters were ligated for hybridization. AMPure XP system (Cat. No. A63881, Beckman Coulter, Indianapolis, IN, USA) purified library fragments to obtain 250~300 bp cDNA. Then, 3 μL USER enzyme (Cat. No. M5505S, Ipswich, MA, NEB, USA) was added to size-selected, adapter-ligated cDNA and incubated sequentially at 37 °C/15 min and 95 °C/5 min for PCR. PCR was performed with Phusion high-fidelity polymerase and universal and Index (X) primers. PCR products were repurified by AMPure XP system, and library quality was evaluated via Agilent 2100 Bioanalyzer (Agilent Technologies, Inc., Santa Clara, CA, USA). After verification, clustering was performed with TruSeq PE Cluster Kit v3-cBot-HS (Illumina, San Diego, CA, USA). Once clustering was completed, sequencing was performed on the Illumina platform. Following sequencing, a series of bioinformatics analyses were implemented, including quality control of raw data, sequence alignment to the reference genome, transcriptome assembly, estimation of gene expression levels, and differential expression analysis. Differentially expressed genes (DEGs) were screened using the criteria of |log2Fold Change| ≥ 1 and FDR < 0.05. Detailed information on biological replicates, clean data volume and genome alignment rate of each sample is summarized in Supplementary Tables S8–S11.

### 4.8. PageMan Analysis

Four comparison groups (T2 vs. T1, Q2 vs. Q1, T2 vs. Q1, Q2 vs. T1) had their log_2_ fold change values imported into PageMan, which enabled the comparison of over-representation among all treatments as described by Usadel [56]. PageMan statistical analysis was employed to predict the significant impacts of BINs, with data analyzed via the Wilcoxon test; BINs were considered significantly different when *p* < 0.05. Blue indicates the enrichment of down-regulated genes, while red indicates the enrichment of up-regulated genes.

### 4.9. Weighted Gene Co-Expression Network Analysis (WGCNA)

To screen gene co-expression modules associated with seed development under different ecological conditions, we performed weighted gene co-expression network analysis (WGCNA) separately on transcriptome datasets of pod husks and seeds using the WGCNA v1.71 package in R Studio (v4.3.1). Genes with FPKM ≥ 1 were retained, and those with low expression variance were filtered out for subsequent network construction. Following the scale-free network criterion, the optimal soft threshold power was determined via the pickSoftThreshold (v1.63-1) function based on the scale-free topology fit index (*R*^2^ > 0.6) and mean connectivity (Figure S2B,D). A signed network was then constructed, and co-expression modules were divided by dynamic tree cutting (Figure S2A,C). Modules with highly correlated eigengenes were merged. Hub genes were screened according to the criteria of module membership (MM > 0.8) and gene significance (GS > 0.8) related to key agronomic traits including grain dry weight and wrinkling rate. Finally, modules significantly correlated with physiological indices were visualized using Cytoscape (v3.9.1) [57].

### 4.10. qRT-PCR Validation

Total RNA was extracted from approximately 0.1 g of pod husk or seed samples using Trizol reagent (Cat. No. 11752050, Invitrogen, Foster City, CA, USA). Sampling was conducted during the grain-filling stage, with 3 biological replicates set for each treatment. To avoid genomic DNA interference, the isolated RNA was processed with DNase I. RNA concentration and purity were assessed by NanoDrop ND-1000 UV-Vis spectrophotometer (Waltham, MA, USA). Reverse transcription was performed with 2.5 μg RNA using Hifair III First-Strand cDNA Synthesis SuperMix (Cat. No. R423-01, Yesen, Nanjing, China). qPCR was run on ABI QuantStudio 6 Real-Time PCR System (Foster City, CA, USA) with Takara SYBR Green PCR Master Mix (Cat. No. RR420A, Takara Bio Inc., Kusatsu, Shiga, Japan); primers are in Supplementary Table S12. The qPCR conditions were as follows: initial denaturation at 95 °C for 10 min, followed by 40 cycles of 95 °C (15 s) denaturation and 60 °C (30 s) extension. Each sample had three technical replicates to reduce variation, and relative expression was quantified by the 2^−ΔΔCT^ method.

### 4.11. Data Analysis and Statistical Methods

SPSS software (IBM SPSS Statistics 26) was used for analysis of variance (ANOVA), Duncan’s multiple comparison test (*p* < 0.05), and Pearson correlation coefficient analysis of physiological data. R language (v4.1.0) with DESeq2, ggplot2, and dynamicTreeCut packages (v1.63-1), as well as Origin 2021 (v9.8), were used for transcriptome data analysis and visualization.

## 5. Conclusions

**Figure 11:**
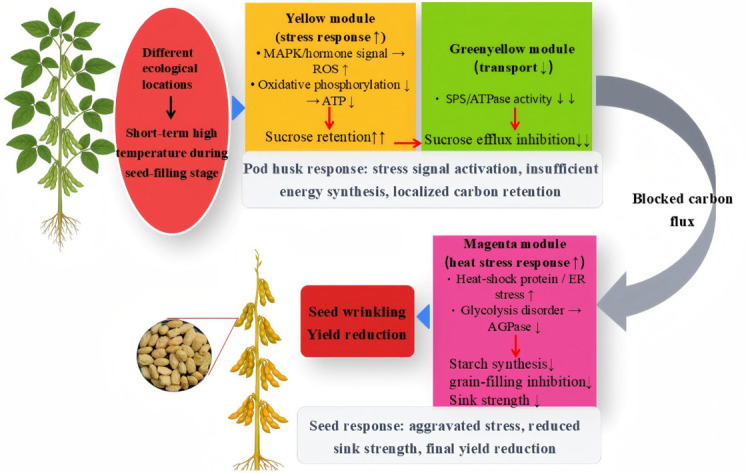
A proposed model of high-temperature-induced seed wrinkling regulated by source–sink–flow energy coupling during seed filling. The black upward (↑) and downward (↓) arrows within the yellow, green-yellow and magenta boxes denote significant upregulation and downregulation of metabolites, respectively; double upward arrows (↑↑) and double downward arrows (↓↓) stand for extremely significant upregulation and downregulation. Solid red straight arrows represent causal regulatory pathways, while the grey curved arrow indicates the feedback loop of blocked carbon flux.

In summary, to explore the formation mechanism of differences in soybean seed wrinkling among different ecological locations, we adopted a comprehensive analysis method combining phenotypic traits, physiological determination, and transcriptomics. Soybean seed wrinkling was dominated by the cultivar × environment interaction effect: temperature differences at the grain-filling stage among ecological locations were the primary driving factor, and the genetic characteristics of cultivars determined the response threshold—HH43 was a high-temperature-sensitive cultivar, while HN76 was a tolerant one. The core physiological mechanism of seed wrinkling was the functional abnormalities of “flow” (pod husks) and “sink” (seeds) in the source–sink–flow system, rather than insufficient source strength. Specifically, it manifested as obstructed sucrose transport and energy synthesis in pod husks and reduced starch synthesis efficiency in seeds. At the molecular level, the disordered expression of genes related to energy metabolism and sucrose transport in pod husks, as well as genes related to cell cycle and starch synthesis in seeds of HH43 under short-term high-temperature conditions at the grain-filling stage in Shenyang, was the fundamental cause of seed wrinkling. WGCNA-identified core modules and hub genes involved in pod husk sucrose transport, heat-shock proteins, and grain starch synthesis serve as key genetic resources to dissect the regulatory mechanisms of soybean grain development and high-temperature response (Figure 11).

## Data Availability

RNA-Seq data have been submitted to the NCBI Sequence ReadArchive (BioProject PRJNA1199776 and BioProject PRJNA1391573).

## Supplementary Materials

The following supporting information can be downloaded at: https://www.mdpi.com/article/10.3390/plants15121924/s1, Figure S1: GO and KEGG enrichment of key module genes in pod husks and seeds; Figure S2: WGCNA analysis of transcriptome data from seed coat and seed samples. (A): Gene dendrogram and co-expression module clustering (pod husk); (B): Determination of the optimal soft threshold for network construction (pod husk); (C): Gene dendrogram and co-expression module clustering (seed); (D): Determination of the optimal soft threshold for network construction (seed). In panels B, D, the left plots show the scale-free topology model fit index (R2) across different soft thresholds, with the red line indicating the R2 = 0.6 threshold; the right plots show mean gene connectivity under each soft threshold; Figure S3: Principal component analysis (PCA) of transcriptome samples. (A): Pod husk samples; (B): Seed samples. Each dot represents one biological replicate. The first two principal components (PC1 and PC2) are shown, with their corresponding variance explained percentages. Samples from the same group are closely clustered, indicating good reproducibility, while different groups are clearly separated, reflecting distinct transcriptomic profiles under different ecological conditions; Table S1: Multivariate analysis of variance (ANOVA) and variation factor contribution rate for wrinkle rate of soybean seeds; Table S2: Two-way analysis of variance (ANOVA) for temperature traits at different growth stages; Table S3: Comparison of partial correlation coefficient and Pearson correlation coefficient between grain wrinkle rate and meteorological factors at grain-filling stage; Table S4: Full-factor multiple regression analysis of grain wrinkle rate of HH43 at grain-filling stage; Table S5: Stepwise regression analysis of grain wrinkle rate of HH43 at grain-filling stage; Table S6: All gene IDs, functional annotations and expression characteristics within each key module; Table S7: The basic soil fertility at the three experimental sites; Table S8: Basic statistics of transcriptome sequencing of soybean pusk samples; Table S9: Transcriptome sequencing and reference genome alignment of soybean pusk samples; Table S10: Basic statistics of transcriptome sequencing of soybean seed samples; Table S11: Transcriptome sequencing and reference genome alignment of soybean seed samples; Table S12: The primers used in qRT-PCR.
