# Supplementary material for: Variations in Ecological Locations Induce Soybean Seed Wrinkles by Disrupting Source–Sink Relationship and Energy Metabolism at the Grain-Filling Stage
Source: Plants (Basel). 2026 Jun 22;15(12):1924. doi: 10.3390/plants15121924 (PMC13306430; doi:10.3390/plants15121924)
Supplement: Supplementary file 1 [file plants-15-01924-s001.zip › plants-4363715-supplementary.pdf]

Table S1 Multivariate analysis of variance (ANOVA) and variation factor contribution rate for wrinkled rate of soybean seeds

Source of variations	df	SS	MS	F-value	P-value	Significance	Contribution rate (%)
Cultivar (C)	1	5521.36	5521.36	2986.45	<0.001	***	29.63
Ecological locations (E)	2	9432.75	4716.38	2546.82	<0.001	***	50.62
Year (Y)	1	128.62	128.62	69.58	0.003	**	0.69
C×E	2	3415.88	1707.94	922.35	<0.001	***	18.33
C×Y	1	15.72	15.72	8.47	0.006	**	0.08
E×Y	2	32.86	16.43	8.92	0.001	**	0.18
C×E×Y	2	22.45	11.23	6.05	0.008	**	0.12
Error	36	66.98	1.86	-	-	-	0.36
Total	47	18636.52	-	-	-	-	100.00

Note : * P < 0.001 (extremely significant), P < 0.01 (significant), * P < 0.05 (significant); the contribution rate of variation factor = (sum of squares of a variation source / total sum of squares)×100, reflecting the ability of this factor to explain the variation of wrinkled rate.

Table S2 Two-way analysis of variance (ANOVA) for temperature traits at different growth stages

Experimental Factors		Sowing-Pod Setting（R3）		Grain-Filling Stage（R5）		Mature Stage（R8）	
		ADT (°C)	DTmax (°C)	ADT (°C)	DTmax (°C)	ADT (°C)	DTmax (°C)
Location	Jiamusi	19.35b	24.70b	20.66b	25.66c	15.61b	21.51b
	Gongzhuling	21.67a	25.11a	24.12a	28.46b	19.45a	24.98a
	Shenyang	22.25 a	25.79a	25.71a	30.83a	21.82a	26.58a
Cultivar	Henong76	20.79	25.23	23.17	28.23b	18.7	24.03
	Heihe43	20.82	25.26	23.2	29.19b	18.73	24.04
Mean squares (ANOVA)	Location (df = 2)	64.03 **	320.76 **	20.16 **	86.08 ***	46.08 **	194.04 **
	Cultivar (df = 1)	ns	ns	ns	12.87**	ns	ns
	Location × Cultivar (df = 2)	ns	ns	ns	2.61**	ns	ns

Note: Letters indicate statistical significance at 0.05 level within the same column. ns indicates p ⩾0.05; ** indicates p < 0.01; *** indicates p < 0.001. The mean square error (MS error) is provided for readers to verify the F-values.

Table S3 Comparison of partial correlation coefficient and Pearson correlation coefficient between grain wrinkled rate and meteorological factors at grain filling stage

Meteorological factors	Correlation coefficient（r）	Partial correlation coefficient（r_p）	Standard error（SE）	t-value	P-value	Significance
DTmax	0.991	0.928	0.042	22.09	<0.001	***
Tmax≥30d	0.975	0.905	0.051	17.75	<0.001	***
DTmin	0.963	0.652	0.123	5.3	0.002	**
ADRH	−0.975	−0.213	0.205	1.04	0.321	ns
ADEP	−0.953	−0.187	0.211	0.89	0.392	ns
ADWV	0.289	0.156	0.218	0.71	0.491	ns
ADTR	−0.832	−0.124	0.223	0.56	0.585	ns

Table S4 Full factor multiple regression analysis of grain wrinkled rate of HH43 at grain filling stage

Independent	Coefficient	Standard error	Standardized coefficient(β)	*P*-value	VIF
constant	−92.47	9.86	-	<0.001	-
DTmax	2.21	0.32	0.58	<0.001	3.26
Tmax≥30d	1.35	0.28	0.4	<0.001	3.26
DTmin	−0.42	0.51	−0.07	0.435	4.78
ADT	0.18	0.63	0.03	0.776	6.85
ADTR	0.15	0.32	0.03	0.651	2.17
ADRH	−0.06	0.05	−0.06	0.261	1.89
EAT	0.04	0.05	0.04	0.443	5.23
ADEP	−0.01	0.02	−0.02	0.63	1.65
ADSD	−0.21	0.31	−0.04	0.514	1.52

Table S5 Stepwise regression analysis of grain wrinkled rate of HH43 at grain filling stage

Model	After adjustment R^2	AIC-value	*F*-value	*P*-value
Wrinkled rate=−82.63+3.27×DTmax	0.923	38.65	215.37	P<0.001
Wrinkled rate =−76.24+2.15×DTmax+1.32×Tmax≥30d	0.955	31.28	178.62	P<0.001
Wrinkled rate =−75.89+2.13×DTmax+1.31×Tmax≥30d−0.04×ADRH	0.953	33.17	115.29	P<0.001

Table S6 All gene IDs, functional annotations and expression characteristics within each key module

Module	Gene symbol	Locus ID	Functional annotation
Yellow	*MAPK/ROS*	Glyma.12G073000; Glyma.06G144500; Glyma.13G095600; Glyma.05G213300; Glyma.11G192700; Glyma.15G155700; Glyma.06G144500; Glyma.12G073000; Glyma.13G102100; Glyma.04G197400; Glyma.02G232600; Glyma.07G105700; Glyma.01G052300; Glyma.08G204800; Glyma.15G087800; Glyma.02G161700; Glyma.05G213300; Glyma.13G035900; Glyma.13G095600; Glyma.05G013300; Glyma.07G198600; Glyma.11G192700; Glyma.20G050800; Glyma.15G155700; Glyma.09G174200; Glyma.01G171100; Glyma.03G022600; Glyma.12G064300	MAPK/ROS Response
Green-yellow	*ATP/SUT*	Glyma.06G154500; Glyma.13G142100; Glyma.02G163200; Glyma.02G270300; Glyma.04G030100; Glyma.16G156900; Glyma.16G157000; Glyma.06G175200; Glyma.15G049200; Glyma.03G142200; Glyma.10G096400; Glyma.05G128900; Glyma.19G134700; Glyma.01G382000; Glyma.07G184300; Glyma.12G056700; Glyma.08G291100; Glyma.01G245900; Glyma.02G064000; Glyma.15G108200; Glyma.13G076500	NADPH oxidase/ROS response/ATPase-coupled transmembrane transport /Mitochondrial tricarboxylic acid (TCA) cycle
Magenta	*AGPaseL1/ HSP*	Glyma.05G037700; Glyma.08G064400; Glyma.04G093200; Glyma.11G150800; Glyma.13G041600; Glyma.14G026100; Glyma.17G195400; Glyma.02G116700; Glyma.06G207400	Heat-shock protein binding/endoplasmic reticulum protein processing
Purple	*GGT/GPX*	Glyma.13G160100; Glyma.08G294300; Glyma.01G170200; Glyma.05G040300; Glyma.01G428400; Glyma.05G196700; Glyma.10G123100	Glutathione peroxidase (GPX)/oxidative homeostasis regulation

Table S7 The basic soil fertility at the three experimental sites

Experimental sites	Total Nitrogen ($g\cdot kg^{-1}$)	Soil Organic Matter ($g\cdot kg^{-1}$)	Available Phosphorus ($mg\cdot kg^{-1}$)	Available Potassium ($mg\cdot kg^{-1}$)
Jiamusi	1.22	18.17	25.57	160.38
Gongzhuling	1.51	19.40	28.70	169.20
Shenyang	1.28	12.15	35.30	151.78

Table S8 Basic statistics of transcriptome sequencing of soybean pusk samples

Sample	Raw Reads	Clean Reads	Clean Base	GC Content (%)	Q20 (%)	Q30 (%)
T1-1	61473850	58765042	8.81	42.86	97.93	93.3
T1-2	43773896	41147788	6.17	44.81	97.56	92.94
T1-3	61171852	55581768	8.34	43.08	97.69	92.72
T2-1	43825124	42465050	6.37	44.67	97.47	92.32
T2-2	54885234	51167792	7.68	43.76	97.8	93.02
T2-3	49091544	46533192	6.98	43.95	97.78	92.87
Q1-1	53115140	51339096	7.7	44.15	97.75	93.77
Q1-2	45644254	43967940	6.6	44.63	97.58	92.82
Q1-3	45506994	43459636	6.52	44.39	97.5	93.21
Q2-1	47691328	45939502	6.89	43.93	97.35	92.64
Q2-2	46105252	43310134	6.5	44.44	97.64	93.48
Q2-3	48971990	47068504	7.06	44.44	97.56	93.42

Table S9 Transcriptome sequencing and reference genome alignment of soybean pusk samples

Sample	Total Reads	Reads mapped	+' mapped	-' mapped	Mapped rate(%)
T1-1	58765042	56360988	28846291	28854923	95.91
T1-2	41147788	40288588	20656804	20664910	97.91
T1-3	55581768	53474107	26534021	26544079	96.21
T2-1	42465050	41325109	19419308	19435758	97.31
T2-2	51167792	49307895	21174196	21187272	96.37
T2-3	46533192	45205404	19285979	19300260	97.15
Q1-1	51339096	49416591	19377726	19389735	96.26
Q1-2	43967940	41249587	19292244	19302260	93.82
Q1-3	43459636	41698991	19530628	19541308	95.95
Q2-1	45939502	44620268	19606142	19614638	97.13
Q2-2	43310134	41951919	18446472	18454621	96.86
Q2-3	47068504	45257565	19423453	19431016	96.15

Table S10 Basic statistics of transcriptome sequencing of soybean seed samples

Sample	Raw Reads	Clean Reads	Clean Base	GC Content (%)	Q20 (%)	Q30 (%)
T1-1	46216844	43707862	6.56	45.68	97.66	93.08
T1-2	47697222	45242156	6.79	45.96	97.78	93.30
T1-3	46866228	44128528	6.62	46.38	97.65	93.02
T2-1	51589850	48992612	7.35	44.95	98.03	94.00
T2-2	45777658	44079018	6.61	46.11	97.65	92.99
T2-3	59172330	57688912	8.65	44.98	98.19	94.28
Q1-1	51714894	49693174	7.45	46.34	97.67	93.07
Q1-2	56902772	55160480	8.27	46.29	97.75	93.27
Q1-3	47472942	45171988	6.78	45.74	97.66	93.07
Q2-1	49222722	47363348	7.10	46.29	97.69	93.12
Q2-2	49212768	46342742	6.95	46.18	97.76	93.26
Q2-3	52401466	50326122	7.55	46.63	97.82	93.44

Table S11 Transcriptome sequencing and reference genome alignment of soybean seed samples

Sample	Total Reads	Reads mapped	+' mapped	-' mapped	Mapped rate (%)
T1-1	43707862	42360988	18846291	18854923	96.91
T1-2	45242156	43288588	17656804	17664910	95.68
T1-3	44128528	42474107	19534021	19544079	96.25
T2-1	48992612	47325109	19419308	19435758	96.60
T2-2	44079018	42307895	21174196	21187272	95.98
T2-3	57688912	55205404	19285979	19300260	95.69
Q1-1	49693174	47416591	19377726	19389735	95.42
Q1-2	55160480	53249587	19292244	19302260	96.54
Q1-3	45171988	43698991	19530628	19541308	96.74
Q2-1	47363348	45620268	19606142	19614638	96.32
Q2-2	46342742	44951919	18446472	18454621	96.70
Q2-3	50326122	49257565	19423453	19431016	97.79

Table S12 The primers used in qRT-PCR

Gene Name	Forward primer (5'to3')	Reverse primer (5'to3')
Actin	ATCTTGACTGAGCGTGGTTATTCC	GCTGGTCCTGGCTGTCTCC
Glyma.06G144500	TGTTCAACACTCTGCACCGA	GCACCTTAATGTCCCCGGAT
Glyma.12G073000	GAAGGCTGTTATGAATGAAACTTTG	TCACGTGCCTTCTGTCATTTTC
Glyma.16G156900	GGCCAAGGTTTATCTTTGGGAGTC	CAAGTTGCCACCACCAAACAAAG
Glyma.03G142200	GAAAGCGTTATTGCTCCAACAGTG	TCCTCAGGTTGTTGTTGATCTTGC
Glyma.05G037700	TGCTTTGATTCGAGCGCAGA	TGCCAAGCTGCTATGACTGG
Glyma.13G160100	TTGGATGTGATACTTCGCCAGTG	TCCCATCCCAACTTGAATTTCCAC
Glyma.08G294300	CCCATCAACCGAACTGAACTGGTG	ACCCACTGTAAGCTCCCAATTTCC
Glyma.01G428400	TGATTTCACGCGGGCCTAAC	GCTCCGAGAGTGATAGCTGGT

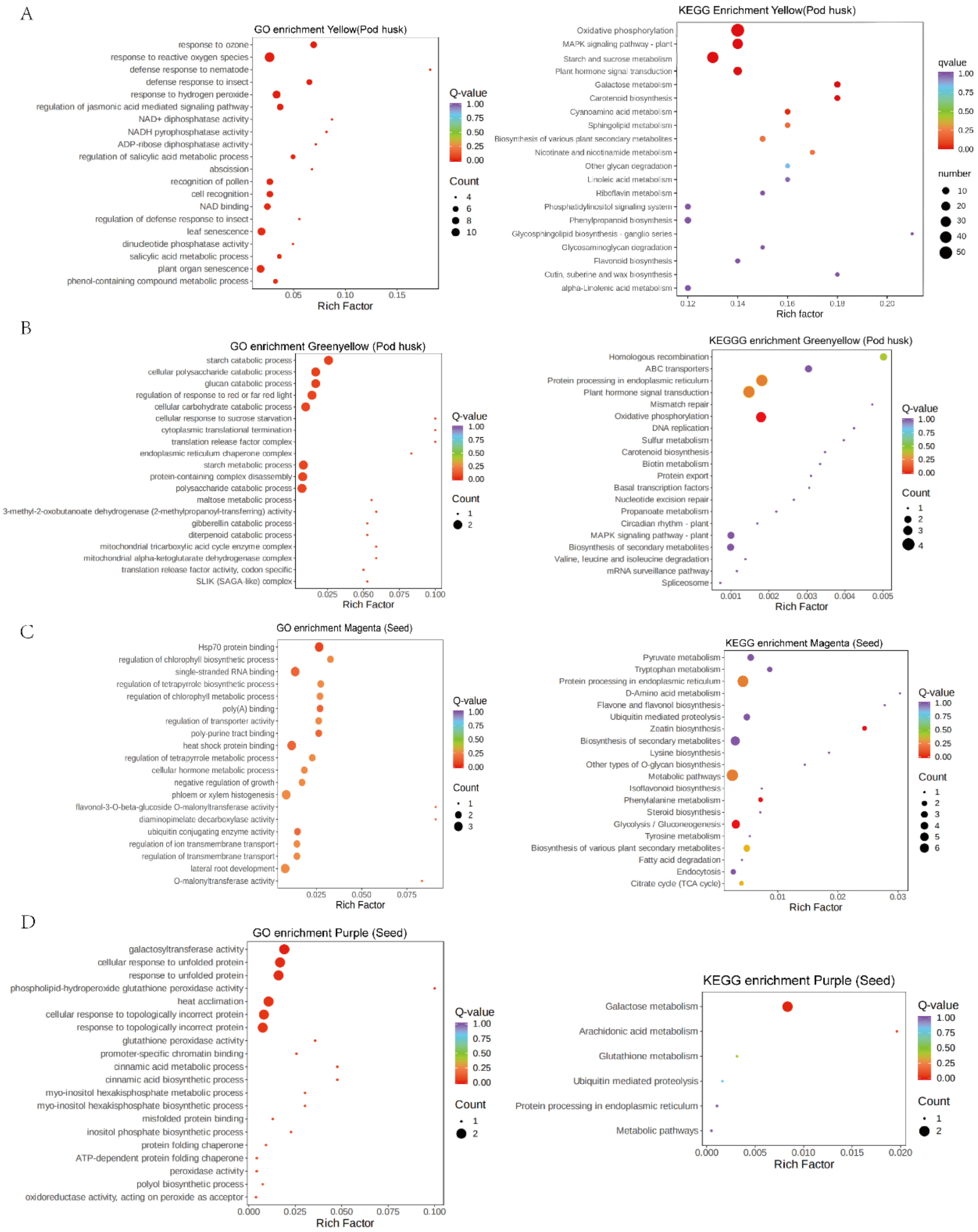

Figure S1 GO and KEGG Enrichment of Key Module Genes in Pod husks and Seeds

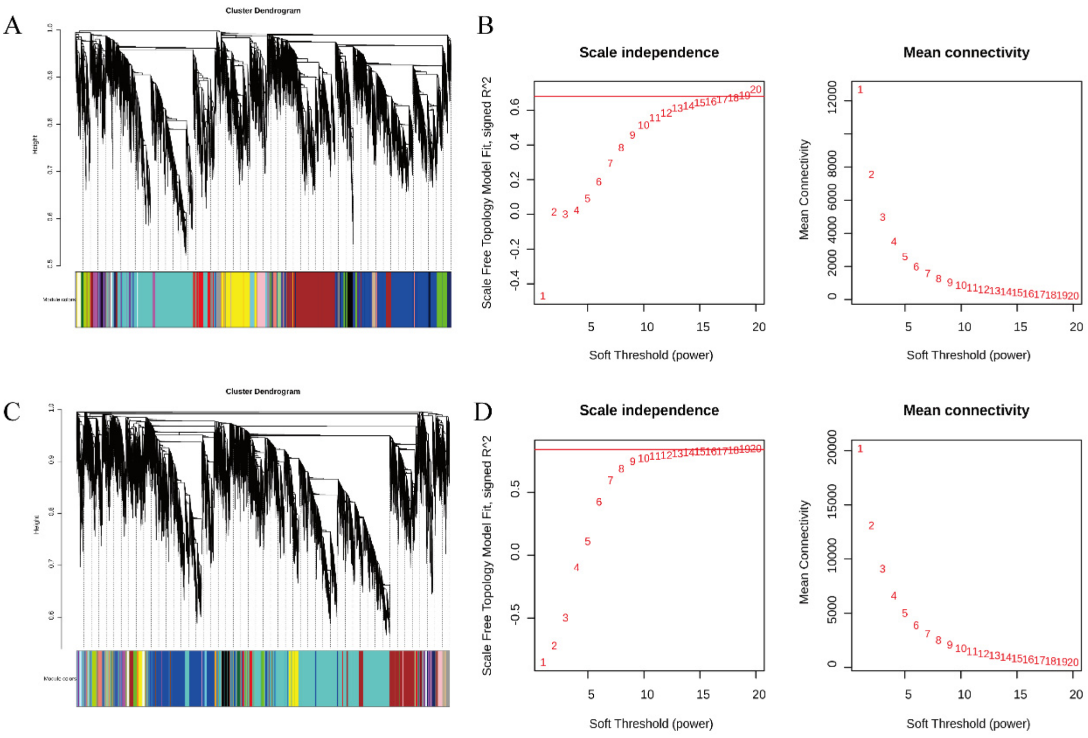

Figure S2 WGCNA analysis of transcriptome data from seed coat and seed samples. **A:** Gene dendrogram and co-expression module clustering (pod husk); **B:** Determination of the optimal soft threshold for network construction (pod husk); **C:** Gene dendrogram and co-expression module clustering (seed); **D:** Determination of the optimal soft threshold for network construction (seed). In panels B, D, the left plots show the scale-free topology model fit index (R^2) across different soft thresholds, with the red line indicating the R^2=0.6 threshold; the right plots show mean gene connectivity under each soft threshold.

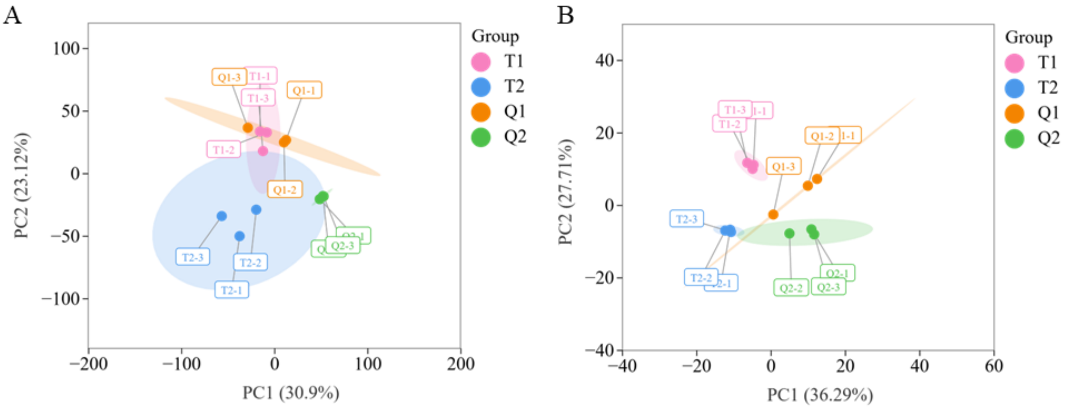

Figure S3 Principal component analysis (PCA) of transcriptome samples. **A:** Pod husk samples; **B:** Seed samples. Each dot represents one biological replicate. The first two principal components (PC1 and PC2) are shown, with their corresponding variance explained percentages. Samples from the same group are closely clustered, indicating good reproducibility, while different groups are clearly separated, reflecting distinct transcriptomic profiles under different ecological conditions.
